# Functional Reconstruction of a Huge Lateral Thoraco‐Abdominal Defect With Combined Innervated Pedicled Latissimus Dorsi Flap and Vastus Lateralis Free Functional Muscle Transfer: A Case Report

**DOI:** 10.1002/micr.70038

**Published:** 2025-02-18

**Authors:** Beniamino Brunetti, Marco Morelli Coppola, Fiorella Oliveri, Valeria Petrucci, Omar Al‐Hilli, Sergio Valeri, Rossana Alloni, Irene Giovanna Aprile, Stefania Tenna, Paolo Persichetti

**Affiliations:** ^1^ Operative Research Unit of Plastic, Reconstructive and Aesthetic Surgery Fondazione Policlinico Universitario Campus Bio‐Medico Rome Italy; ^2^ Department of Medicine and Surgery, Research Unit of Plastic, Reconstructive and Aesthetic Surgery Università Campus Bio‐Medico di Roma Rome Italy; ^3^ Unit of Breast Surgery Ospedale Isola Tiberina—Gemelli Isola Rome Italy; ^4^ Department of Plastic and Maxillofacial Surgery Uppsala University Hospital Uppsala Sweden; ^5^ Operative Research Unit of Soft‐Tissue Sarcomas Surgery Fondazione Policlinico Universitario Campus Bio‐Medico Rome Italy; ^6^ Operative Research Unit of Specialized General Surgery Fondazione Policlinico Universitario Campus Bio‐Medico Rome Italy; ^7^ Research Unit of Specialized General Surgery, Department of Medicine and Surgery Università Campus Bio‐Medico di Roma Rome Italy; ^8^ Department of Neuromotor Rehabilitation IRCCS Fondazione Don Carlo Gnocchi Onlus Florence Italy

**Keywords:** abdominal wall, free tissue flaps, muscle strength, sarcoma, thoracic wall

## Abstract

Full thickness thoraco‐abdominal defects are traditionally challenging to reconstruct, exposing the patient to a significant risk of wound complications and functional impairment. The authors present an extremely challenging and unique case where a huge lateral thoraco‐abdominal defect was reconstructed with a combination of innervated flaps allowing to completely restore contour and function of the operated region. A 77‐year‐old male patient presented with a leiomyosarcoma arising in the right lateral thoraco‐abdominal region. Full‐thickness resection involved the anterior part of the latissimus dorsi (LD) and serratus anterior muscles along with IX to XII ribs, a cuff of diaphragm muscle and the entire lateral abdominal wall, creating a 30 × 25 cm defect with exposure of right lung, liver, and ascending and transverse colon. After the placement of a synthetic mesh, a 28 × 16 cm pedicled innervated LD flap was advanced in V‐Y fashion to cover the thoracic part of the defect. Then the patient was turned supine and a vastus lateralis free functional muscle transfer (FFMT) from the contralateral thigh was used to reconstruct the abdominal part of the defect. The recipient vessels were provided transposing the ipsilateral deep inferior epigastric pedicle according to the extra‐anatomical pedicle rerouting technique. Motor branch for the VL was coapted to a sizeable intercostal nerve. The muscle was covered with split‐thickness skin grafts. Both flaps healed uneventfully, allowing to obtain a complete restoration of form and function with M5 score according to the MRC Scale for muscle strength 8 months after surgery. Functional reconstruction of the lateral abdominal wall with FFMT has never been reported. Our successful case shows the importance of functional reconstruction of lateral thoraco‐abdominal defects to prevent abdominal weakness or herniation, trunk instability, postural deficiencies and core strength loss.

## 1 Introduction

Reconstruction of combined defects involving both thoracic and abdominal walls, either thoraco‐abdominal or thoraco‐lumbar, represents a significant challenge for plastic surgeons (Brunetti, Marchica, et al. 2022). A full‐thickness wall defect, with violation of muscular and osteo‐cartilaginous planes, not only determines the immediate exposure of inner organs, with an increased risk of life‐threatening complications, such as herniation and infection, but also causes a significant long‐term functional impairment, making the contraction of abdominal and respiratory muscles impossible and leading to trunk instability, paradoxical respiratory movements, postural deficiencies, core strength loss, and massive eventration (Kapur and Butler 2018). In such cases, the reconstructive plan should aim at restoring as much form and function as possible. Nevertheless, flap reconstruction in these peculiar anatomical regions is made particularly challenging by several factors. Potentially expendable loco‐regional flaps such as latissimus dorsi (LD), including its reverse variation, and antero‐lateral thigh (ALT) myocutaneous or fasciocutaneous flaps present a limited reach not able to cover defects in higher abdominal and lumbar regions (Kotti et al. 2012; Winter et al. 2019; Cammarata et al. 2024). In alternative, free flaps are the only reliable solution, despite the absence of adequate recipient vessels in close proximity to the defect and the need to resort to vein grafts or arteriovenous loops to accomplish the reconstruction (Baumann and Butler 2012; Gurunluoglu et al. 2016; Brunetti, Petrucci, Tenna, et al. 2024). The evidence about the superiority of functional reconstruction is poor, with most of the authors focusing on functional reconstruction of abdominal wall central defects (Patel et al. 2018). On the other hand, to date there are no reports available in the literature about total functional reconstructive approach in complex lateral‐thoraco‐abdominal wall defects involving both chest and lateral abdominal wall.

In this report, the authors present an extremely challenging and unique case where a huge lateral thoraco‐abdominal defect was reconstructed with a combination of innervated flaps allowing to completely restore contour and function of the operated region, describing for the first time in the literature the application of a free functional muscle transfer (FFMT) for a lateral abdominal wall defect.

## 2 Case Report

**FIGURE 1:**
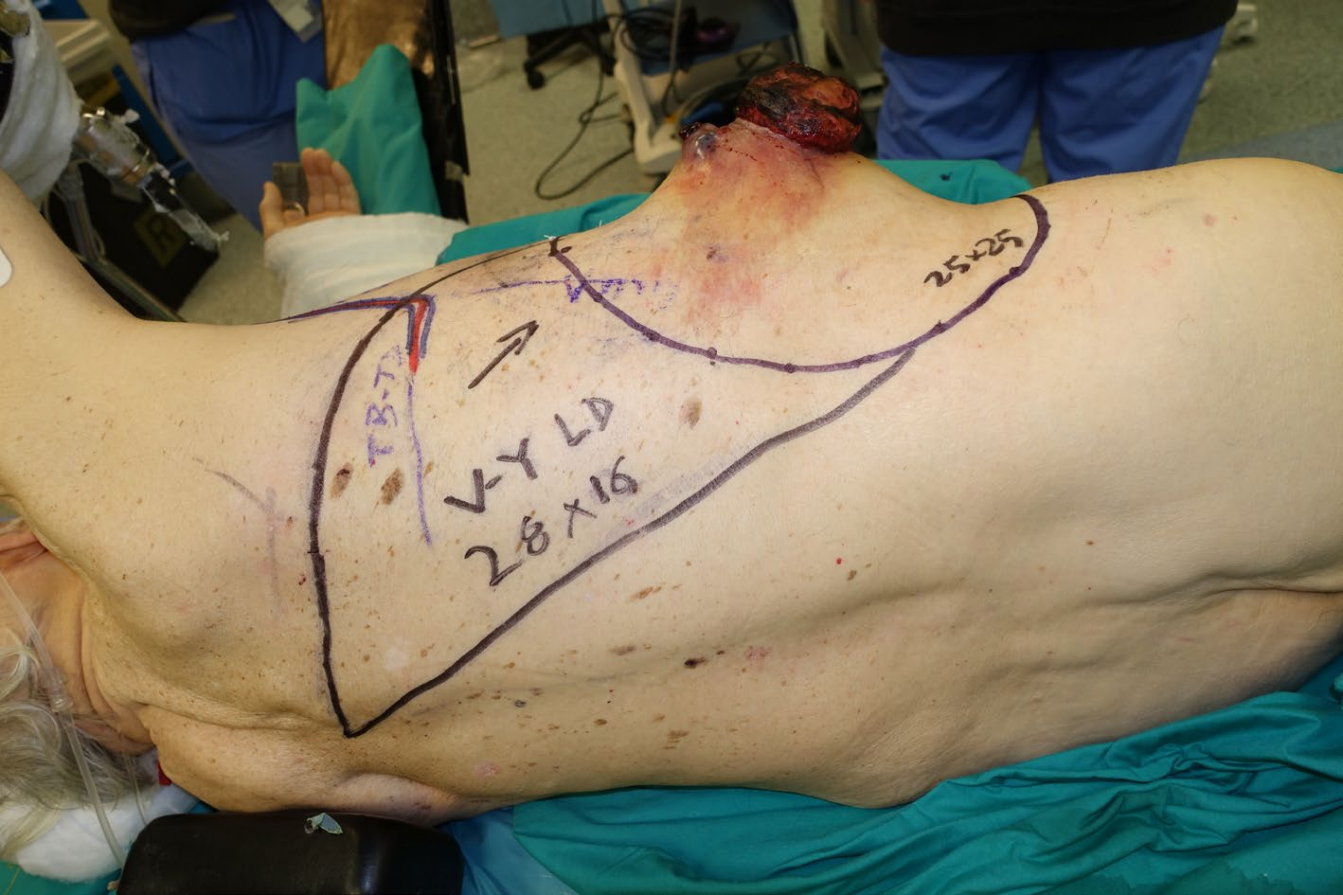
Patient affected by ulcerated leiomyosarcoma of the lateral thoraco‐abdominal wall.

**FIGURE 2:**
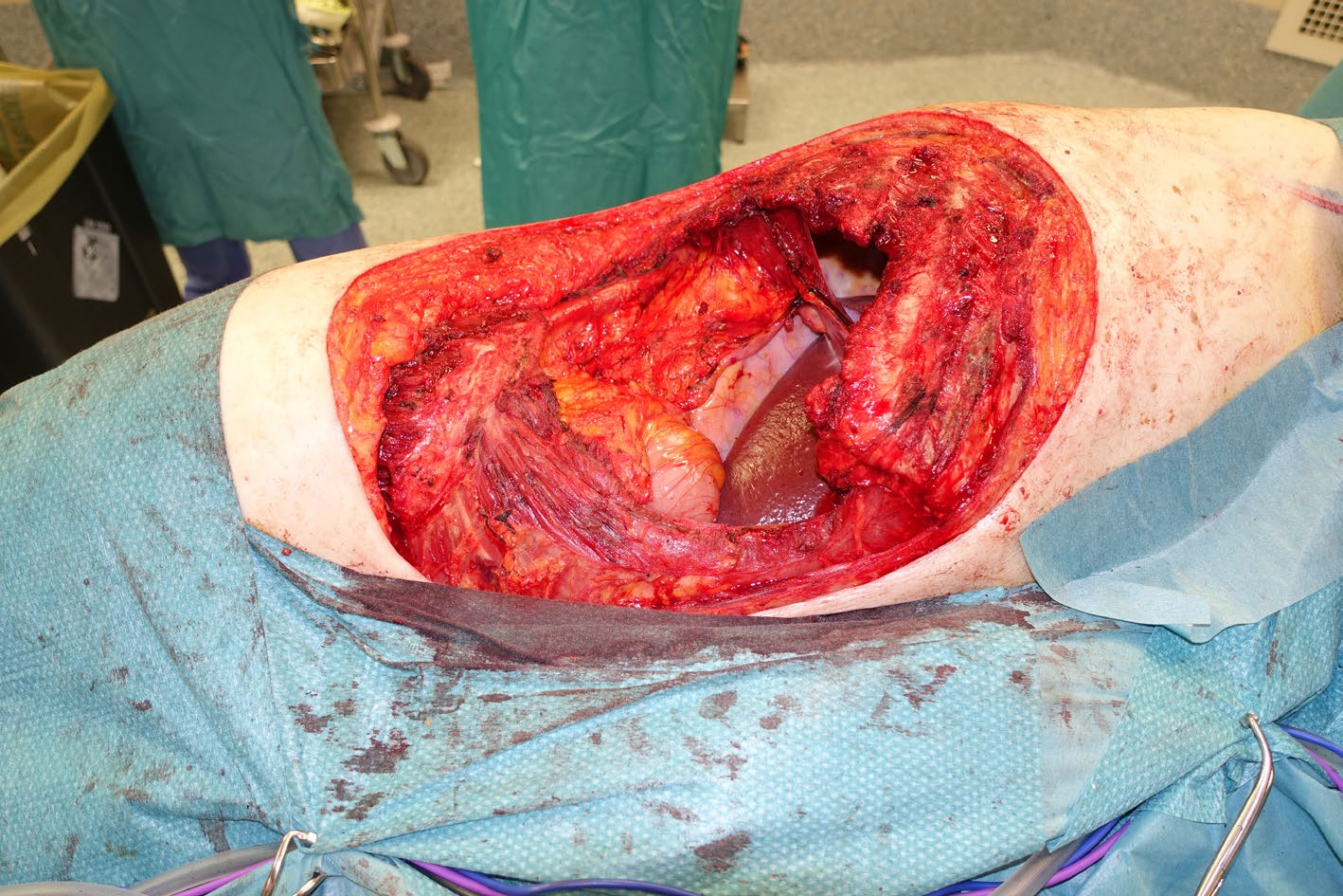
The defect after tumor resection.

**FIGURE 3:**
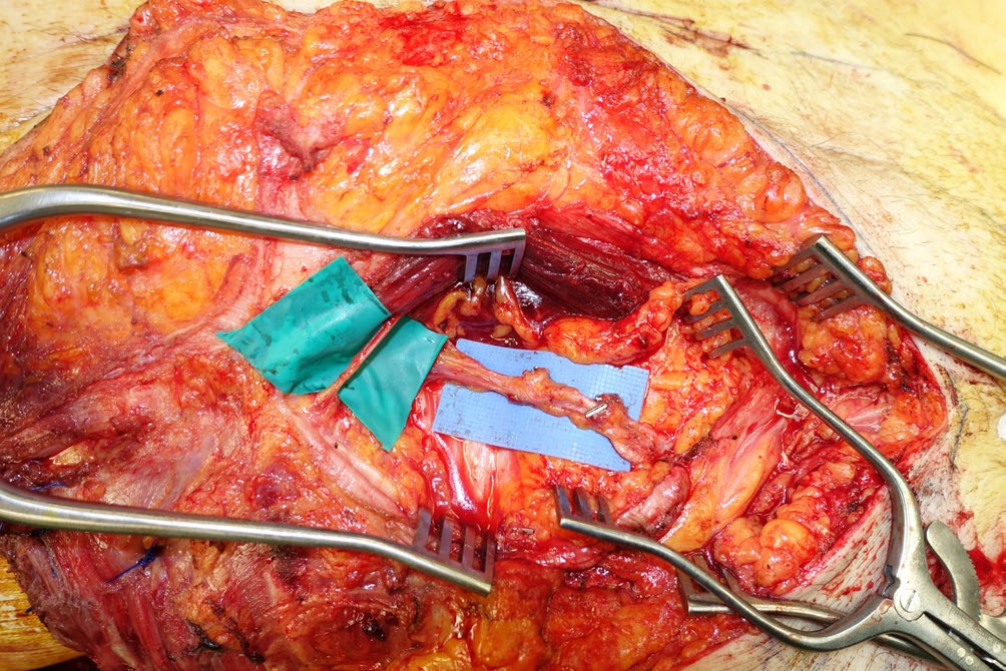
The right deep inferior epigastric vessels are transposed toward the lumbar to be used as recipient vessels for vastus lateralis free functional muscle transfer. An intercostal nerve is selected for nerve coaptation.

A 77‐year‐old male patient presented to the Soft‐Tissue Sarcomas Unit of our Institution with a huge sarcoma arising in the right lateral thoraco‐abdominal region (Figure 1). Three months earlier, he had previously undergone partial resection of this lesion, whose dimensions were 5.5 × 4 cm and whose histopathological diagnosis was high‐grade sarcoma. The re‐evaluation of histological sample performed at our hospital was suggestive of leiomyosarcoma. A multidisciplinary evaluation of the case was scheduled at the Institutional sarcoma board. Magnetic resonance imaging (MRI) showed a 10‐cm mass involving the lateral abdominal wall, extending to the external oblique, the internal oblique, and the transversus abdominis muscles with dislocation of the peritoneal fascia. A total‐body CT scan ruled out distant metastases. Due to the rapid progression of the disease and the high risk of bleeding and ulceration, the patient was referred for upfront surgery. Full‐thickness resection, performed in lateral decubitus, involved the anterior part of the LD and serratus anterior muscles along with the IX to XII ribs, a cuff of diaphragm muscle, and the entire lateral abdominal wall, creating a 30 × 25 cm defect with exposure of right lung, liver, and ascending and transverse colon (Figure 2). The diaphragmatic defect was minimal and managed by direct plication to maintain a proper intrathoracic pressure. A 25 × 20 cm GORE DUALMESH Biomaterial (W.L. Gore and Associates, Flagstaff, AZ) was used to provide visceral coverage. The synthetic mesh was fixed using interrupted 2–0 polypropylene sutures: anteriorly to the fascia of the rectus abdominis muscle, inferiorly to the iliac crest, superiorly to the residual ribs, and posteriorly to the remaining fascia of the broad abdominal muscles. The defect was found to be too extensive to be reconstructed with a single flap, thus requiring a combination of two flaps to enable reliable full coverage. A 28 × 16 cm pedicled innervated LD flap was advanced in V‐Y fashion to cover the thoracic part of the defect. The flap's humeral insertion and nerve supply were preserved to reduce the risk of thoracic herniation and respiratory impairment (Tenna et al. 2020). The donor site of the flap was closed primarily. The patient was then turned supine, and the full‐thickness lateral abdominal wall defect was reassessed. Therefore, a neurotized ALT‐VL myocutaneous free flap harvested from the contralateral thigh was chosen to reconstruct the abdominal part of the defect. Intra‐operatively, the flap had to be converted to a muscle flap because only a single antero‐medial thigh perforator was found to nourish the skin of the thigh, precluding the use of the designed skin island, which was instead utilized as a source of skin grafts to finalize the reconstruction. Due to the absence of reliable vascular pedicles near the defect, recipient vessels for the flap were provided transposing the ipsilateral deep inferior epigastric artery and vein (DIEA‐V) from the lower abdomen toward the right lumbar, according to the extra‐anatomical pedicle rerouting technique (Brunetti, Petrucci, Tenna, et al. 2024) (Figure 3). A 7‐cm long DIEA‐V pedicle loop was sufficient to perform straightforward anastomoses and flap inset. The motor branch to the VL muscle was coapted to a sizeable intercostal nerve with an end‐to‐end epi‐perineural neurorrhaphy. The muscular continuity of the lateral abdominal and thoraco‐lumbar regions was restored by the suturing the VL muscle to the mobilized LD flap posteriorly and to the lateral margin of rectus abdominis muscle anteriorly, obtaining complete coverage of the mesh. The VL muscle was covered with split‐thickness skin grafts derived from its own overlying skin. The immediate post‐operative course was uneventful until the ninth post‐operative day, when the patient developed a small bowel herniation across the mesh and the VL flap, requiring re‐exploration through a midline laparotomy and placement in the retro‐muscular space of a second mesh, medial to the first one, to strengthen the posterior fascia of the reconstructed abdominal wall. Except for this complication, both flaps showed no signs of vascular compromise, allowing for complete healing of the reconstructed site 6 weeks after revision surgery.

The patient was instructed to wear abdominal compressive garments for 3 months. Two weeks after the first surgery, the patient started a two‐month rehabilitation program, involving two 50‐min sessions every day, 6 days a week. During the first 2 weeks, the patient performed transfers (without weight on the donor limb that was protected by a brace), sit trunk control exercises, joint mobility exercises and muscle strengthening of the upper limbs and of the right lower limb; between the third and the fourth week, exercises on the donor limb were added to recover range of motion of the knee and muscle strength by isometric contractions of the extensor muscles of the left knee; verticalization and loading activities on the donor limb were introduced cautiously after 20 days from the surgery. At the end of 2 months of the rehabilitation treatment, the patient could perform transfers and postural transitions without help, walk without aids, and take the stairs with alternating steps and no support. Muscle strength was recovered in both lower limbs. The patient was independent in all activities of daily living. Following rehabilitation, he demonstrated significant improvements in functional independence, with modified Barthel index (mBI) score increasing from 40 to 95, and in walking ability, assessed by the Adapted Patient Evaluation Conference System (APECS) score, improved from 0 to 7 (Galluccio et al. 2024).

**FIGURE 4:**
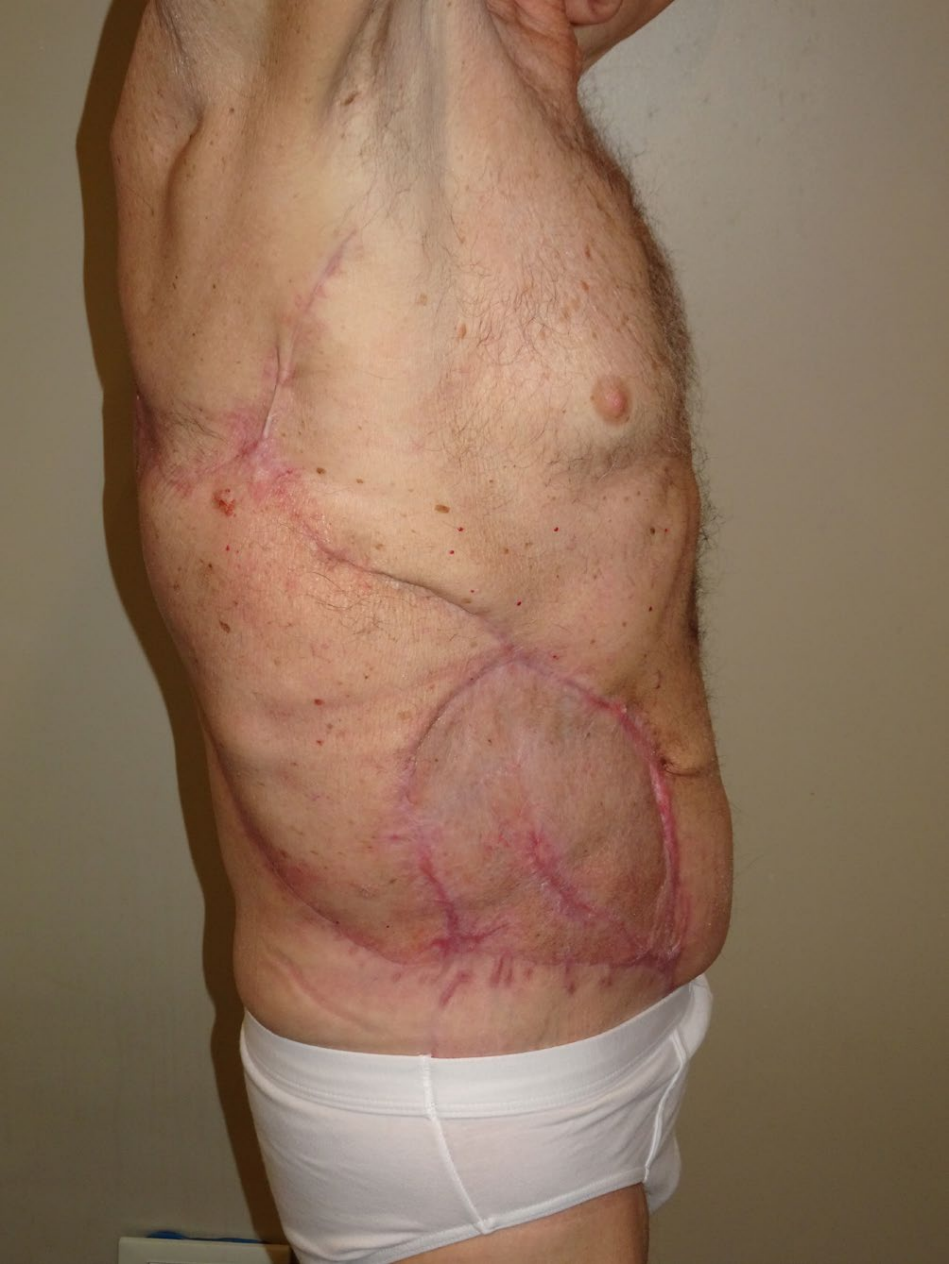
Eight months postoperative: Good abdominal and thoraco‐dorsal wall shape.

Eight months after the procedure, optimal restoration the thoraco‐abdominal contour was observed, with adequate abdominal tone at rest, in the absence of signs of hernia in both standing and supine positions (Figure 4). Furthermore, neurotization of both muscles was confirmed with clinical examination showing voluntary contraction of the transplanted muscles, with the patient able to strengthen the lateral abdominal wall and perform abdominal crunches against gravity, therefore, reaching a M5 score according to the Medical Research Council (MRC) scale for muscle strength. The full case is illustrated in the Video S1.

The patient was referred for close clinical and radiological follow‐up. The first post‐operative total‐body CT scan, performed 3 months after surgery, was negative, but the second one, performed 6 months post‐operatively, detected multiple, bilateral, lung metastases. The patient was subsequently referred for chemotherapy with doxorubicin (75 mg/m^2^) and dacarbazine (800 mg/m^2^) (D'Ambrosio et al. 2020).

## 3 Discussion

Complex thoraco‐abdominal defects are rarely encountered in the clinical practice, but when they do occur, they pose a significant risk of life‐threatening respiratory and abdominal complications. Most of the literature about abdominal wall reconstruction focuses about the type of mesh to choose in combination with the different available soft tissue flaps, but solid evidence about which patients would benefit from functional reconstruction is still lacking (Heller et al. 2012; Mathes et al. 2000; Song et al. 2018; Brunetti et al. 2020; Brunetti, Morelli Coppola, et al. 2022; Brunetti, Morelli Coppola, Petrucci, et al. 2024). In this scenario, few solutions to address functional reconstruction of abdominal wall defects have been reported. Ninkovic described the use of free innervated LD muscle flap; Brunetti described a modification of the classical LD flap to recruit more skin for extensive reconstructions (Ninković et al. 1998; Ninkovic et al. 2022; Brunetti et al. 2023). Koshima reported cases of dynamic reconstruction by means of free neurotized rectus femoris muscle flap, according to like‐with‐like strategy (Koshima et al. 1999, 2003). Iida addressed the issue with free innervated VL combined with ALT free flap, while Vranckx adopted pedicled innervated VL and ALT (PIVA) flap with the same purpose (Iida et al. 2013; Vranckx et al. 2015). All these papers described a functional approach for massive defects of the central abdominal wall, being well known that resection of a single rectus abdominis muscle can be easily managed with a mesh‐only approach without requirements of FFMT. On the other hand, isolated defects of the lateral abdominal wall or combined lateral thoraco‐abdominal defects are more rarely encountered in the clinical practice and less literature is available about their management (Baumann and Butler 2012). Lateral abdominal wall is composed of different muscular layers responsible for abdominal continency and, most important, represents the anatomical region from where intercostal nerves emerge to reach the rectus abdominis muscles and guarantee contraction and continency of the central abdominal wall (Pulikkottil et al. 2015). Therefore, full thickness resection of the lateral abdominal wall represents the worst‐case reconstructive scenario, being the abdominal continency put at risk both directly (through muscle resection) and indirectly (through denervation of the rectus abdominis muscle). For these reasons, massive full‐thickness resections of the lateral abdominal wall may be a clear indication for providing patients with functional reconstruction. There is no clear evidence supporting the superiority of a combined approach compared to a functional flap without a mesh. However, most authors advocate for the use of a mesh, except in cases of contaminated wounds. Ninkovic reported one case of abdominal wall weakness, in which a mesh was not used in the primary reconstruction and had to be added in a second surgery (Ninkovic et al. 2022). The use of a mesh delivers extra support to the reconstruction, especially during the temporary denervation of FFMT, which require a long recovery time, until reinnervation occurs. Pedicled innervated locoregional flaps or free muscular flaps with intact innervation have no temporary impairment, but the behavior of a FFMT will be more natural in terms of muscle inset and restoration of physiological muscle voluntary contraction.

Our functional reconstructive approach was based on a combination of two innervated flap. The V‐Y advancement innervated LD flap was selected to guarantee not only coverage of the thoracic part of the defect with primary closure of the donor site but also to provide thoracic stabilization against paradoxical respiratory movements, being the LD flap an accessory respiratory muscle.

When the patient was moved to supine position, a pedicled ipsilateral ALT‐VL flap was deemed too risky to safely cover the high abdominal part of the defect. Necrosis of the distal portion of the flap could have resulted in mesh exposure and subsequent failure of the reconstruction. A pedicled innervated rectus femoris would have not covered the entire residual defect. Moreover, in high‐grade sarcoma reconstruction, the use of pedicled flaps should be limited given the high risk of local relapse and donor‐site seeding.

A contralateral LD flap with L‐shaped or kiss skin paddle configuration would have been a reliable option but would have forced us to change the patient's position twice (Brunetti et al. 2023; Brunetti, Salzillo, et al. 2022). Considering that the VL had already proven effective as a FFMT in restoring knee extension function after resection of sarcomas of the quadriceps, a neurotized VL free flap harvested from the contralateral thigh was chosen to reconstruct the abdominal part of the defect (Brunetti, Morelli Coppola, Tenna, et al. 2024; Brunetti et al. 2025).

The poor availability of reliable recipient vessels in the lumbars may preclude the use of free flaps in these regions, but the EPR technique allowed us to overcome this limitation (Brunetti, Petrucci, Tenna, et al. 2024).

While nerve supply to the LD muscle flap was maintained, obtaining an immediate stabilization of the chest wall, complete reinnervation of the VL muscle was observed 8 months post‐operatively. Unfortunately, the patient was diagnosed with systemic disease progression, which precluded further long‐term follow‐up appointments and the execution of EMG to objectively confirm muscle neurotization. Additionally, the single‐case nature of our experience and the subsequent lack of statistical validation of our findings constitute further limitations. Despite these, we still believe that the management of such complex and rarely encountered cases deserve the attention of the plastic surgery readership. Our successful case shows the importance of functional reconstruction of complex lateral thoraco‐abdominal defects to stabilize the chest wall and prevent abdominal weakness or herniation, postural deficiencies, and core strength loss. More studies will be needed to confirm the long‐term efficacy and stability of such complex reconstructions.

## Ethics Statement

This study did not involve animals. All procedures performed involving human participants were in accordance with the ethical standards of the institution and with the 1964 Helsinki Declaration and its later amendments. Institutional Review Board approval for data collection was obtained. All participants gave written informed consent.

## Conflicts of Interest

The authors declare no conflicts of interest.

## Supporting information


**Video S1.** Supporting Information.

## Data Availability

The data that support the findings of this study are available from the corresponding author upon reasonable request.

## References

[micr70038-bib-0001] Baumann, D. P. , and C. E. Butler . 2012. “Lateral Abdominal Wall Reconstruction.” Seminars in Plastic Surgery 26, no. 1: 40–48. 10.1055/s-0032-1302465. 23372458 PMC3348741

[micr70038-bib-0002] Brunetti, B. , M. Barone , S. Tenna , R. Salzillo , F. Segreto , and P. Persichetti . 2020. “Pedicled Perforator‐Based Flaps: Risk Factor Analysis, Outcomes Evaluation and Decisional Algorithm Based on 130 Consecutive Reconstructions.” Microsurgery 40, no. 5: 545–552. 10.1002/micr.30590. 32298004

[micr70038-bib-0003] Brunetti, B. , P. Marchica , M. Morelli Coppola , et al. 2022. “Extended Latissimus Dorsi Flap With Propeller Ascending Design for Reconstruction of a Complex Lateral Lumbar Defect: A Case Report and Review of the Literature.” Microsurgery 42, no. 4: 366–371. 10.1002/micr.30842. 34796966

[micr70038-bib-0004] Brunetti, B. , M. Morelli Coppola , S. Ciarrocchi , R. Salzillo , S. Tenna , and P. Persichetti . 2022. “"Thou Shalt Not Throw Away a Living Thing": Innovative Use of Perforator Flaps in Abdominal Wall Reconstruction.” Plastic and Reconstructive Surgery 150, no. 3: 672–676. 10.1097/PRS.0000000000009450. 35789148

[micr70038-bib-0005] Brunetti, B. , M. Morelli Coppola , R. De Bernardis , et al. 2025. “Chimeric Anterolateral Thigh‐Vastus Lateralis Free Flap With Propeller Skin Island for Functional Quadriceps Reconstruction.” Journal of Hand and Microsurgery 17, no. 2: 100200. 10.1016/j.jham.2024.100200. 39816724 PMC11730876

[micr70038-bib-0006] Brunetti, B. , M. Morelli Coppola , V. Petrucci , et al. 2024. “From Angiosomal to bi‐Angiosomal and Extra‐Angiosomal Pedicled Perforator Flaps: Optimizing the Use of Local Tissues in Abdominal Wall Reconstruction.” Microsurgery 44, no. 6: e31229. 10.1002/micr.31229. 39258388

[micr70038-bib-0007] Brunetti, B. , M. Morelli Coppola , S. Tenna , et al. 2024. “Thigh Reconstruction Between Form and Function: An Algorithm for Flap Selection Based on a Series of 70 Oncological Patients.” Microsurgery 44, no. 1: e31121. 10.1002/micr.31121. 37799094

[micr70038-bib-0008] Brunetti, B. , V. Petrucci , S. Tenna , et al. 2024. ““Extra‐Anatomical Pedicle Rerouting” an Alternative Technique to Obtain New Recipient Vessels for Microsurgical Reconstruction in Unfavorable Clinical Situations.” Journal of Plastic, Reconstructive and Aesthetic Surgery 91: 227–235. 10.1016/j.bjps.2024.01.055. 38428230

[micr70038-bib-0009] Brunetti, B. , R. Salzillo , S. Tenna , et al. 2022. “Total Autologous Breast Reconstruction With the Kiss Latissimus Dorsi Flap.” Journal of Plastic, Reconstructive and Aesthetic Surgery 75, no. 10: 3673–3682. 10.1016/j.bjps.2022.06.078. 36055926

[micr70038-bib-0010] Brunetti, B. , R. Salzillo , S. Tenna , et al. 2023. “Abdominal Wall Reconstruction With the Free Functional L‐Shaped Latissimus Dorsi Flap: A Case Report.” Microsurgery 43, no. 6: 617–621. 10.1002/micr.31070. 37226360

[micr70038-bib-0011] Cammarata, E. , F. Toia , M. Maltese , M. Rossi , M. Tripoli , and A. Cordova . 2024. “Soft Tissue Reconstruction of the Trunk With Pedicled Perforator and Musculocutaneous Flaps: A Single‐Center Comparative Retrospective Study.” Microsurgery 44, no. 1: e31131. 10.1002/micr.31131. 38009980

[micr70038-bib-0012] D'Ambrosio, L. , N. Touati , J. Y. Blay , et al. 2020. “Doxorubicin Plus Dacarbazine, Doxorubicin Plus Ifosfamide, or Doxorubicin Alone as a First‐Line Treatment for Advanced Leiomyosarcoma: A Propensity Score Matching Analysis From the European Organization for Research and Treatment of Cancer Soft Tissue and Bone Sarcoma Group.” Cancer 126, no. 11: 2637–2647. 10.1002/cncr.32795. 32129883

[micr70038-bib-0013] Galluccio, C. , M. Germanotta , S. Valeri , et al. 2024. “Soft Tissue Sarcoma With Lower Limb Impairment: Development of a Specific Rehabilitation Protocol Based on Demolitive and Reconstructive Surgery Types.” Journal of Clinical Medicine 13, no. 23: 7023. 10.3390/jcm13237023. 39685483 PMC11642672

[micr70038-bib-0014] Gurunluoglu, R. , A. Ghaznavi , D. Krpata , J. Zins , and M. J. Rosen . 2016. “Arteriovenous Loop Graft in Abdominal Wall Reconstruction Using Free Tissue Transfer.” Journal of Plastic, Reconstructive and Aesthetic Surgery 69, no. 11: 1513–1515. 10.1016/j.bjps.2016.09.005. 27667547

[micr70038-bib-0015] Heller, L. , C. Chike‐Obi , and A. S. Xue . 2012. “Abdominal Wall Reconstruction With Mesh and Components Separation.” Seminars in Plastic Surgery 26, no. 1: 29–35. 10.1055/s-0032-1302463. 23372456 PMC3348745

[micr70038-bib-0016] Iida, T. , M. Mihara , M. Narushima , et al. 2013. “Dynamic Reconstruction of Full‐Thickness Abdominal Wall Defects Using Free Innervated Vastus Lateralis Muscle Flap Combined With Free Anterolateral Thigh Flap.” Annals of Plastic Surgery 70, no. 3: 331–334. 10.1097/SAP.0b013e3182321b64. 22214798

[micr70038-bib-0017] Kapur, S. K. , and C. E. Butler . 2018. “Lateral Abdominal Wall Reconstruction.” Seminars in Plastic Surgery 32, no. 3: 141–146. 10.1055/s-0038-1666801. 30046290 PMC6057781

[micr70038-bib-0018] Koshima, I. , T. Moriguchi , K. Inagawa , and K. Urushibara . 1999. “Dynamic Reconstruction of the Abdominal Wall Using a Reinnervated Free Rectus Femoris Muscle Transfer.” Annals of Plastic Surgery 43, no. 2: 199–203. 10454330

[micr70038-bib-0019] Koshima, I. , Y. Nanba , T. Tutsui , Y. Takahashi , S. Itoh , and R. Kobayashi . 2003. “Dynamic Reconstruction of Large Abdominal Defects Using a Free Rectus Femoris Musculocutaneous Flap With Normal Motor Function.” Annals of Plastic Surgery 50, no. 4: 420–424. 10.1097/01.SAP.0000032304.45784.F6. 12671387

[micr70038-bib-0020] Kotti, B. , O. Jaidane , J. Ben Hassouna , and K. Rahal . 2012. “The “Reverse” Latissimus Dorsi Flap for Large Lower Lumbar Defect.” Case Reports in Surgery 2012: 964625. 10.1155/2012/964625. 23082273 PMC3469085

[micr70038-bib-0021] Mathes, S. J. , P. M. Steinwald , R. D. Foster , W. Y. Hoffman , and J. P. Anthony . 2000. “Complex Abdominal Wall Reconstruction: A Comparison of Flap and Mesh Closure.” Annals of Surgery 232, no. 4: 586–596. 10.1097/00000658-200010000-00014. 10998657 PMC1421191

[micr70038-bib-0022] Ninković, M. , P. Kronberger , C. Harpf , A. Rumer , and H. Anderl . 1998. “Free Innervated Latissimus Dorsi Muscle Flap for Reconstruction of Full‐Thickness Abdominal Wall Defects.” Plastic and Reconstructive Surgery 101, no. 4: 971–978. 10.1097/00006534-199804040-00013. 9514329

[micr70038-bib-0023] Ninkovic, M. , M. Ninkovic , D. Öfner , and M. Ninkovic . 2022. “Reconstruction of Large Full‐Thickness Abdominal Wall Defects Using a Free Functional Latissimus Dorsi Muscle.” Frontiers in Surgery 9: 853639. 10.3389/fsurg.2022.853639. 35372467 PMC8968006

[micr70038-bib-0024] Patel, N. G. , I. Ratanshi , and E. W. Buchel . 2018. “The Best of Abdominal Wall Reconstruction.” Plastic and Reconstructive Surgery 141, no. 1: 113–136. 10.1097/PRS.0000000000003976. 29280882

[micr70038-bib-0025] Pulikkottil, B. J. , R. A. Pezeshk , L. N. Daniali , S. H. Bailey , S. Mapula , and R. E. Hoxworth . 2015. “Lateral Abdominal Wall Defects: The Importance of Anatomy and Technique for a Successful Repair.” Plastic and Reconstructive Surgery—Global Open 3, no. 8: e481. 10.1097/GOX.0000000000000439. 26495194 PMC4560214

[micr70038-bib-0026] Song, Z. , D. Yang , J. Yang , et al. 2018. “Abdominal Wall Reconstruction Following Resection of Large Abdominal Aggressive Neoplasms Using Tensor Fascia Lata Flap With or Without Mesh Reinforcement.” Hernia 22, no. 2: 333–341. 10.1007/s10029-018-1738-8. 29417339 PMC5978915

[micr70038-bib-0027] Tenna, S. , R. Salzillo , B. Brunetti , et al. 2020. “Effects of Latissimus Dorsi (LD) Flap Harvest on Shoulder Function in Delayed Breast Reconstruction. A Long‐Term Analysis Considering the Acromiohumeral Interval (AHI), the WOSI, and BREAST‐Q Questionnaires.” Journal of Plastic, Reconstructive and Aesthetic Surgery 73, no. 10: 1862–1870. 10.1016/j.bjps.2020.05.047. 32586755

[micr70038-bib-0028] Vranckx, J. J. , A. M. Stoel , K. Segers , and L. Nanhekhan . 2015. “Dynamic Reconstruction of Complex Abdominal Wall Defects With the Pedicled Innervated Vastus Lateralis and Anterolateral Thigh PIVA Flap.” Journal of Plastic, Reconstructive and Aesthetic Surgery 68, no. 6: 837–845. 10.1016/j.bjps.2015.03.009. 25964228

[micr70038-bib-0029] Winter, R. , M. Steinböck , W. Leinich , et al. 2019. “The Reverse Latissimus Dorsi Flap: An Anatomical Study and Retrospective Analysis of its Clinical Application.” Journal of Plastic, Reconstructive and Aesthetic Surgery 72, no. 7: 1084–1090. 10.1016/j.bjps.2019.03.010. 30926412

